# A fast algorithm for determining bounds and accurate approximate *p*-values of the rank product statistic for replicate experiments

**DOI:** 10.1186/s12859-014-0367-1

**Published:** 2014-11-21

**Authors:** Tom Heskes, Rob Eisinga, Rainer Breitling

**Affiliations:** Institute for Computing and Information Sciences, Radboud University Nijmegen, Nijmegen, The Netherlands; Department of Social Science Research Methods, Radboud University Nijmegen, Nijmegen, The Netherlands; Manchester Institute of Biotechnology, Faculty of Life Sciences, University of Manchester, Manchester, UK

**Keywords:** Rank product statistic, *p*-value distribution, Transcriptomics, Proteomics, Metabolomics

## Abstract

**Background:**

The rank product method is a powerful statistical technique for identifying differentially expressed molecules in replicated experiments. A critical issue in molecule selection is accurate calculation of the *p*-value of the rank product statistic to adequately address multiple testing. Both exact calculation and permutation and gamma approximations have been proposed to determine molecule-level significance. These current approaches have serious drawbacks as they are either computationally burdensome or provide inaccurate estimates in the tail of the *p*-value distribution.

**Results:**

We derive strict lower and upper bounds to the exact *p*-value along with an accurate approximation that can be used to assess the significance of the rank product statistic in a computationally fast manner. The bounds and the proposed approximation are shown to provide far better accuracy over existing approximate methods in determining tail probabilities, with the slightly conservative upper bound protecting against false positives. We illustrate the proposed method in the context of a recently published analysis on transcriptomic profiling performed in blood.

**Conclusions:**

We provide a method to determine upper bounds and accurate approximate *p*-values of the rank product statistic. The proposed algorithm provides an order of magnitude increase in throughput as compared with current approaches and offers the opportunity to explore new application domains with even larger multiple testing issue. The R code is published in one of the Additional files and is available at http://www.ru.nl/publish/pages/726696/rankprodbounds.zip.

**Electronic supplementary material:**

The online version of this article (doi:10.1186/s12859-014-0367-1) contains supplementary material, which is available to authorized users.

## Background

Post-genomic data analysis (transcriptomics, proteomics, metabolomics) is often concerned with the identification of differentially expressed molecules (transcripts, proteins, metabolites) under different experimental conditions (e.g., treatment vs. control) using multiple biological replicates. A simple and widely used non-parametric statistical method, initially introduced by Breitling *et al*. [1] for gene expression microarrays, is to rank the molecules within each experiment in order of evidence for differential expression and to calculate the product of the ranks across experiments. This rank product method is based on the common biological belief that if a molecule is repeatedly at the top of the lists ordered by up- or down-regulation fold change in multiple treatment–control experiments, the molecule is more likely to be differentially expressed.

The rank product statistic is particularly useful for the analysis of noisy datasets and a small number of replicates, as it does not rely on any distributional assumptions [1–4]. Its main weakness is sensitivity to variations in molecule-specific variance, namely higher variance of weakly expressed molecules. This limitation is mitigated, in practice, by variance-stabilizing normalization [5]. The rank product method is used to combine ranked lists in gene expression profiling and in various other postgenomic datasets with ranked scores, including proteomics and metabolomics [6–8]. Such ranking is important because only a limited number of candidate molecules (transcripts or proteins or metabolites) can usually be followed up in a typical biological downstream analysis for confirmation or further study. Another advantage of the ranking is the resulting suppression of the unwanted influence of correlated behaviour between different molecules. In contrast to traditional marginal tests, such as the *t*-test, in the rank product approach correlating molecules ‘compete’ for positions in the ranked list. In the extreme case of identical behaviour of all molecules, a *t*-test would yield the same (possibly false positive) result for all molecules, whereas in a rank product test, ties in the ranked list would be broken randomly, guaranteeing that none of them would be considered differentially expressed. As a useful side effect of this feature, the rank product test becomes increasingly conservative as larger fractions of the set of molecules studied are differentially expressed: if all molecules are changing to the same extent, their rank ordering will again be random.

Having ranked the molecules by their rank product, the next step is to obtain the *p*-value associated with each molecule under the null hypothesis that the molecule is not differentially expressed in any of the independent replicate experiments. The crux here is the requirement to correct for multiple testing inherent in the need to perform one test per queried molecule. Methods that use the entire distribution of *p*-values to estimate or control the false discovery rate (FDR) assume and perform well only when accurate *p*-values are available [9]. It is therefore imperative to obtain the most accurate probability estimates in applications that involve a massive number of tests [10], such as in the analysis of transcriptome profiling data.

For this reason, exact calculation is preferred in computing *p*-values for use in subsequent molecule-specific FDR-adjustment procedures. Eisinga *et al*. [11] recently provided a derivation of the exact probability distribution of the discrete rank product statistic and its true tail probabilities. An obstacle of exact calculation is that, whereas the *p*-values of small rank products can be calculated swiftly, computing the probabilities of large rank products may consume considerable amounts of time. Although the speed of execution will depend on computing power, exact *p*-value calculation becomes time prohibitive in multiple experiments for rank product values exceeding 10^7^. Unfortunately, in a typical large postgenomic molecular profiling study, such rank products may occur for the bulk of the molecules analysed.

If exact calculation is infeasible, re-sampling-based inference methods such as permutation testing may be considered. The permutation re-sampling procedure involves a trade-off between accuracy and number of permutations [12]. That is, the number of permutations needed is always larger than the inverse of the *p*-value, but a factor of the order of 100 or so more permutations is required so that the *p*-values can be accurately estimated to several decimal places for performing multiple-testing adjustment. In practice, the number of permutation samples may perhaps go up to 10^13^, but re-sampling then starts to become unrealistically expensive, meaning that it is hard to accurately estimate *p*-values smaller than 10^−11^. Such *p*-values are common in rank product analysis of the expression values of many molecules in multiple batches.

As an alternative procedure, Koziol [13] suggested to use the continuous gamma distribution to approximate the sampling distribution of the discrete rank product statistic. For large rank products the gamma calculation performs well, and for extremely large values the gamma *p*-values are close to exact. However, Eisinga *et al*. [11] have shown that for smaller rank product values, i.e., the ones biologists are most interested in, the gamma approximation has a serious bias, overestimating *p*-values by several orders of magnitude, and that the error increases as the *p*-values become smaller.

There is therefore a range of intermediate rank product values in postgenomic studies where current approaches, exact calculation and stochastic and deterministic approximations, all have serious drawbacks in terms of computation time, accuracy or both. The goal of this paper is to obtain guaranteed lower and, in particular, upper bounds for the *p*-values of any rank product value observed, with the conservative upper bound protecting against false positives. The strict bounds may also be exploited to quickly calculate accurate approximate *p*-values for rank product analysis of a variety of postgenomic molecular profiling data.

## Methods

The rank product approach was originally derived for paired experiments (two-colour microarrays). However it can be applied for unpaired data, which are common in postgenomic molecular profiling, by creating random pairs of experiments and calculating the average rank product for several random pairings. Without loss of generality, we thus consider *n* molecules profiled in *k* paired experiments. In each experiment *i*, a molecule receives a random ranking *r*_*i*,_ i.e., any number between 1 and *n.* We define *G*_*k*_(*ρ)*/*n*^*k*^ as the probability that the product of these random rankings is smaller than rank product *ρ*:

$$ {G}_k\left(\rho \right)={\displaystyle \sum_{r_{\;1}=1}^n{\displaystyle \sum_{r_{\;2}=1}^n\dots {\displaystyle \sum_{r_{\;k}=1}^n\varTheta \left(\rho -{r}_1\times {r}_2\times \dots \times {r}_k\right),}}} $$

with the Heaviside step function Θ(*x*) = 1 iff *x* ≥ 0 and 0 otherwise. We obviously have *G*_*k*_(*ρ*) = 0 for any *ρ* < 1 and *G*_*k*_(*ρ*) = *n*^*k*^ for *ρ ≥ n*^*k*^. Our starting point is the observation that the distribution of *ρ* for *k* experiments relates to that for *k −* 1 experiments. Since any rank product *ρ* based on *k* experiments can be written as the product of a rank *r*_1_ in the first experiment times a rank product *ρ*' based on *k −* 1 experiments, we have:

1 $$ {G}_k\left(\rho \right)={\displaystyle \sum_{r_{\;1}=1}^n{G}_{k-1}\left(\rho /{r}_1\right)}={G}_{k-1}\left(\rho \right)+{\displaystyle \sum_{r_{\;1}=2}^{\min \left(\rho, n\right)}{G}_{k-1}\left(\rho /{r}_1\right).} $$

Here the upper limit min(*ρ*,*n*) explicitly incorporates that *ρ*' can never be smaller than 1, so *r*_1_ can never be larger than *ρ*. In theory, we could use this recursion to compute *G*_*k*_(*ρ*). In practice, this is unfeasible for large *ρ, n*, and/or *k*. The discrete nature of this recursion makes it difficult, if not impossible, to obtain a generic analytical solution. However, as we will show below, it is possible to bound and approximate this sum through integrals that can be evaluated analytically.

Our line of reasoning is as follows. Since *G*_*k*_(*ρ*) is a cumulative function, it is monotonically increasing in *ρ*. Sums over monotonically increasing or decreasing functions can be bounded by integrals. In doing so, we turn the discrete recursion (1) involving a summation into continuous recursions involving integrals, one for a lower bound and another one for an upper bound. Recursions involving integrals are typically easier to solve than recursions involving summations. The upper limit min(*ρ*,*n*) in the discrete recursion (1) translates to the same upper limit in the continuous recursion, basically implementing the fact that a rank product *ρ*' based on *k −* 1 experiments cannot contribute to a rank product *ρ* based on *k* experiments if *ρ*' > *ρ*. This upper limit is a highly nonlinear function of *ρ*, which then also does not allow for an easy solution of the continuous recursion. By consistently separating the cases *ρ ≥ n* and *ρ*' *≥ n* from *ρ* < *n* and *ρ*' *< n*, we will see that the solution of the continuous recursions can be written as a piecewise function, with recursions for the separate pieces still in terms of integrals, but now with limits that are linear rather than nonlinear functions of the rank product *ρ.*

Perhaps surprisingly, these recursions for the separate pieces, each corresponding to a different interval for the rank product *ρ*, can be solved analytically. That is, these solutions can be written in terms of basic functions, the parameters of which follow a simple recursion that can be implemented in a fast algorithm. So, in the end, we have managed to turn the complicated recursion (1) on a function *G*_*k*_(*ρ*) into a simple recursion on parameters that specify piecewise continuous upper and lower bounds on *G*_*k*_(*ρ*). The following sections describe the steps in mathematical detail.

### Integral and piecewise recursion

Since any combination of ranks that contributes to *G*_*k*_(*ρ*) also contributes to *G*_*k*_(*ρ*') if *ρ*' ≥ *ρ*, we easily see that *G*_*k*_(*ρ*) is monotonically (not necessarily strictly) increasing in *ρ* for any *k*. But then *G*_*k*−1_(*ρ*/*r*_1_) is monotonically decreasing in *r*_1_. Summations over monotonically decreasing functions can be bounded by integrals (and vice versa). As the following theorem indicates, this can be used to derive upper and lower bounds that obey recursion equations involving integrals instead of summations.

Theorem 1. *Consider the two functions*${\overline{G}}_k\left(\rho \right)$*and*${\underset{\bar{\mkern6mu}}{G}}_k\left(\rho \right)$*that satisfy the recursions*

2 $$ {\overline{G}}_k\left(\rho \right)={\overline{G}}_{k-1}\left(\rho \right)+{\displaystyle {\int}_1^{\min \left(\rho, n\right)}\;{\overline{G}}_{k-1}\left(\rho /r\right)}\;dr $$

*and*

3 $$ {\underset{\bar{\mkern6mu}}{G}}_k\left(\rho \right)={\underset{\bar{\mkern6mu}}{G}}_{k-1}\left( \max \left(1,\rho /n\right)\right)+{\displaystyle {\int}_1^{\min \left(\rho, n\right)}{\underset{\bar{\mkern6mu}}{G}}_{k-1}\left(\rho /r\right)}\;dr, $$

*and are both initialized at*${\overline{G}}_0\left(\rho \right)={\underset{\bar{\mkern6mu}}{G}}_0\left(\rho \right)={G}_0\left(\rho \right)=\varTheta \left(\rho -1\right).$*For any k* ≥ 0 *and ρ* ≤ *n*^*k*^*we have*

$$ {\underset{\bar{\mkern6mu}}{G}}_k\left(\rho \right)\le {G}_k\left(\rho \right)\le {\overline{G}}_k\left(\rho \right). $$

*That is,*${\overline{G}}_k\left(\rho \right)$*gives an upper bound on G*_*k*_(*ρ*) *and*${\underset{\bar{\mkern6mu}}{G}}_k\left(\rho \right)$*a lower bound.* The proof is detailed in Additional file 1.

For ease of exposition, we introduce the constant ∆ and consider the recursion

4 $$ {\tilde{G}}_k\left(\rho \right)=\varDelta {\tilde{G}}_{k-1}\left(\rho \right)+\left(1-\varDelta \right){\tilde{G}}_{k-1}\left( \max \left(1,\rho /n\right)\right)+{\displaystyle {\int}_1^{\min \left(\rho, n\right)}\;{\tilde{G}}_{k-1}\left(\rho /r\right)}\;dr. $$

Setting ∆ to either 0 or 1, we obtain the recursion for the lower and upper bound, respectively. We will argue and empirically show that an accurate approximation (but no guaranteed bound) can be obtained by taking the geometric mean of the upper and lower bound.

The recursion starts from ${\tilde{G}}_0\left(\rho \right)=\varTheta \left(\rho -1\right).$ The constraint that ${\tilde{G}}_k\left(\rho \right)={G}_k\left(\rho \right)=0$ for *ρ* < 1, and the consequence that the upper limit of the integral is a nonlinear function of *ρ*, seriously complicates the solution of the recursion (4). However, we will see that if we write ${\tilde{G}}_k\left(\rho \right)$ as a piecewise function,

5 $$ {\tilde{G}}_k\left(\rho \right)=\left\{\begin{array}{c}\hfill {\tilde{G}}_{k0}\left(\rho \right)={n}^k\;\hfill \\ {}\hfill {\tilde{G}}_{kj}\left(\rho \right)\kern1.44em \hfill \\ {}\hfill\;{\tilde{G}}_{k,k+1}\left(\rho \right)=0\hfill \end{array}\right.\kern0.6em \begin{array}{c}\hfill \mathrm{if}\;\rho\ \ge\ {n}^k\kern5.04em \hfill \\ {}\hfill \kern0.35em \mathrm{if}\;{n}^{k-j}\le \rho <{n}^{k-j+1};\kern0.1em j=1\dots k\hfill \\ {}\hfill \mathrm{if}\ \rho <1,\kern5.4em \hfill \end{array} $$

the recursion equation for the pieces ${\tilde{G}}_{kj}\left(\rho \right)$ simplifies considerably and can in fact be solved.

Theorem 2. *With*${\tilde{G}}_k\left(\rho \right)$*a piecewise function of the form (*5*), the pieces*${\tilde{G}}_{kj}\left(\rho \right)$*satisfy, for* 1 ≤ *j* ≤ *k* − 1, *the recursion*

6 $$ {\tilde{G}}_{kj}\left(\rho \right)=\varDelta {\tilde{G}}_{k-1,j-1}\left(\rho \right)+\left(1-\varDelta \right){\tilde{G}}_{k-1,j}\left(\rho /n\right)+{\displaystyle {\int}_1^{\rho /{n}^{k-j}}dr\;{\tilde{G}}_{k-1,j-1}\left(\rho /r\right)}+{\displaystyle {\int}_{\rho /{n}^{k-j}}^n\;{\tilde{G}}_{k-1,j}\left(\rho /r\right)\;dr,} $$

*and, for j* = *k*,

7 $$ {\tilde{G}}_{kk}\left(\rho \right)=\varDelta {\tilde{G}}_{k-1,k-1}\left(\rho \right)+1-\varDelta +{\displaystyle {\int}_1^{\rho}\;{\tilde{G}}_{k-1,k-1}\left(\rho /r\right)}\;dr. $$

The proof is given in Additional file 2. The intuition behind the piecewise construction follows if one tries to construct the recursion for *k* =1,2,3, and so on. For *k* =1, *ρ* is always smaller than *n*, so max(1, *ρ*/*n*) =1 and min(*ρ*, *n*) = *ρ*. For *k* =2, we can separate the cases *ρ* ≥ *n* and *ρ < n*, corresponding to the pieces ${\tilde{G}}_{21}$ and ${\tilde{G}}_{21}$, respectively. For *k* =3, we again separate the cases *ρ* ≥ *n* and *ρ* < *n*, but now we also have to check whether *ρ*/*r* in the integrand ${\tilde{G}}_2\left(\rho /r\right)$ is larger than *n* (i.e., refers to ${\tilde{G}}_{22}\Big)$ or smaller than *n* (i.e., refers to ${\tilde{G}}_{21}\Big).$ Working this out, one realizes that three different pieces are needed for ${\tilde{G}}_3.$ Induction on *k* leads to the piecewise function (5) and the recursions (6) and (7). These recursions now involve integrals, instead of summations, with limits that are either constants or linear in *ρ*, instead of a nonlinear function of *ρ.*

### Lattice

**Figure 1:**
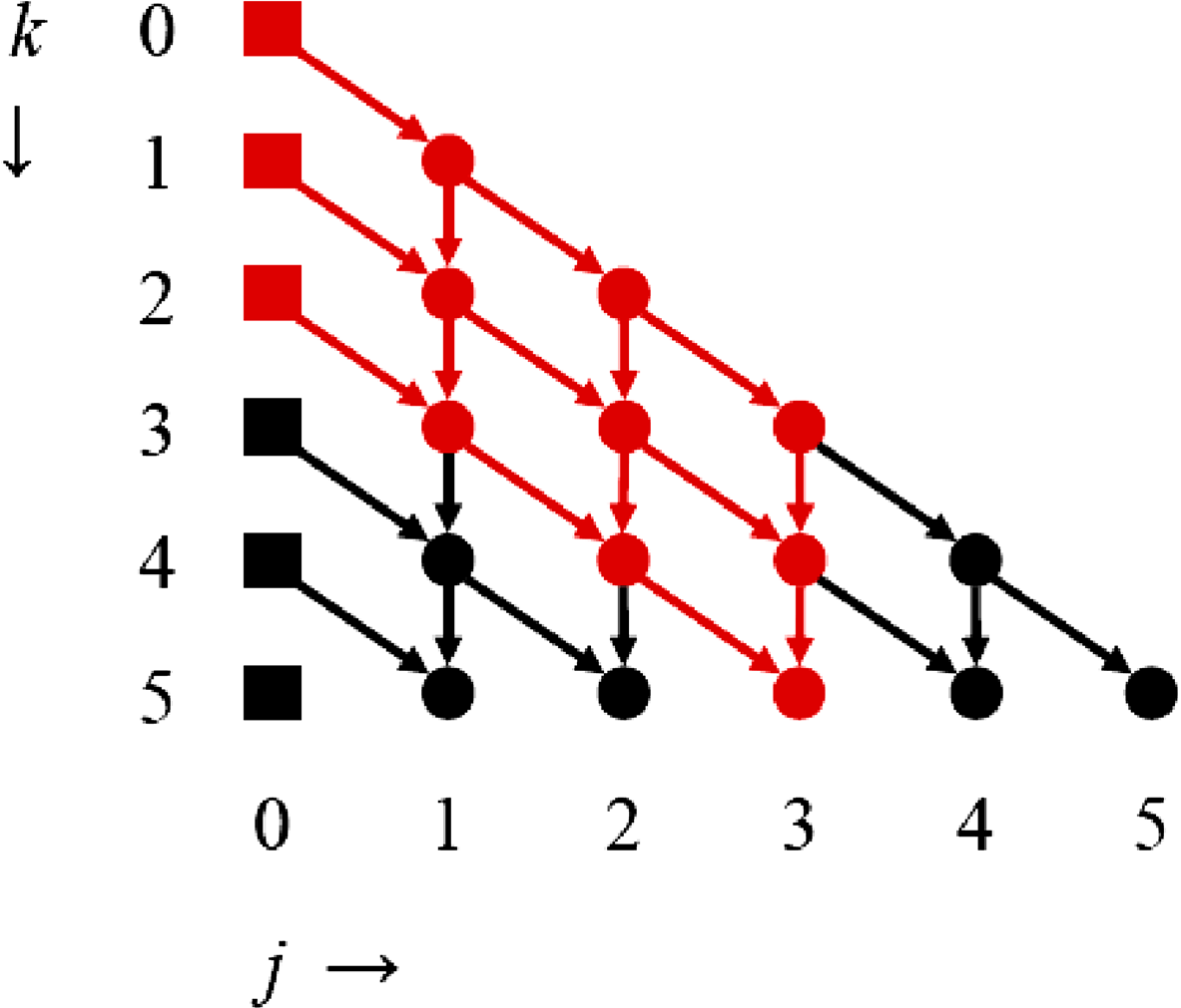
**Visualization of recursion.** Visualization of recursion with *k*, the numbers of experiments, on the *y*-axis, and *j*, the index of the interval that contains the rank product *ρ*, on the *x*-axis. Nodes correspond to combinations of *k* and *j*. The squares are given: ${\tilde{G}}_{k0}\left(\rho \right)={n}^k,$ with *n* the number of molecules. The arrows show the dependencies between the nodes. For example, to compute ${\tilde{G}}_{4,2}\left(\rho \right),$ we first need to compute ${\tilde{G}}_{3,1}\left(\rho \right)$ and ${\tilde{G}}_{3,2}\left(\rho \right)$. The red path visualizes the calculations required to obtain ${\tilde{G}}_{5,3}\left(\rho \right).$

Figure 1 sketches the dependencies between different combinations of *k* and *j,* where *j* is the index of the interval [*n*^*k*−*j*^,*n*^*k*−*j*+1^] that contains the rank product *ρ*, i.e., *j* = ceiling(*k* − log *ρ*/log *n*).

An actual implementation to compute ${\tilde{G}}_{kj}\left(\rho \right)$ can be recursive, e.g., starting at node (*k*,*j*) and recursively computing the parameters that are needed. The alternative is to pre-calculate which parameters are needed and then go through these in two for-loops. To compute ${\tilde{G}}_{kj}\left(\rho \right),$ one possibility is then to have an outer loop with *j*' running from 0 to *j* (from left to right on the lattice in Figure 1), with an inner loop with *k*' running from *j*' to max(*k*, *k* − *j* + *j*') (from top to bottom). The other option is to have an outer loop with *k*' running from 0 to *k* (from top to bottom) and *j*' from max(*k*' − *k* + *j*, 0) to min(*k*', *j*) (from left to right).

### Functional form

The recursions (6) and (7), together with the initialization ${\tilde{G}}_{k\hbox{'}0}\left(\rho \right)={n}^{k\hbox{'}},$ fully determine ${\tilde{G}}_{kj}\left(\rho \right)$ for any *ρ* (and corresponding *j*) and *k*. We could replace analytical integration by numerical integration. However, trying a few steps, one soon realizes that the integrations that are required in each of the steps can be done analytically and a pattern starts to emerge. It appears that every solution can be written in the form

8 $$ {\tilde{G}}_{kj}\left(\rho \right)={\varepsilon}_{kj}+{\delta}_{kj}\rho +{\boldsymbol{\gamma}}_{kj}^T\varPsi \left(\rho; {\boldsymbol{\alpha}}_{kj},{n}^{k-j+{\boldsymbol{\beta}}_{k\ j}}\right), $$

with

$$ \varPsi \left(\rho; \alpha, \lambda \right)\equiv \rho {\left( \log \left[\frac{\rho }{\lambda}\right]\right)}^{\alpha }, $$

and appropriate choices for the parameters ***α***, ***β***, ***γ***, *δ*, and *ε*. Here ***α***_*kj*_, ***β***_*kj*_, and ***γ***_*kj*_ are vectors of equal length. We used vector notation such as

$$ {\gamma}^T\varPsi \left(\rho; \boldsymbol{\alpha}, {n}^{k-j+{\beta}_{kj}}\right)={\displaystyle \sum_m{\gamma}_m\varPsi \left(\rho; {\alpha}_m,{n}^{k-j+{\beta}_m}\right),} $$

where the sum runs over all elements of the vectors.

Theorem 3. *The solutions of the recursions (*6)* and (*7*), starting from the initialization*${\tilde{G}}_{k\hbox{'}0}={n}^{k\hbox{'}},$*can be written in the form (*8*).* See Additional file 3 for the proof.

### Updates and implementation

Now that we have confirmed that the solution is indeed of the form (8), what remains is to find the proper updates of the parameters *θ* ≡ {***α***, ***β***, ***γ***, *δ*, *ε*}. These are given in the following theorem, the proof of which is given in Additional file 4.

Theorem 4. *The parameters θ* ≡ {***α***, ***β***, ***γ***, *δ*, *ε*} *of the solution*${\tilde{G}}_{kj}\left(\rho \right)$*obey the update equations, for* 1 ≤ *j* ≤ *k* − 1,

9 $$ \begin{array}{l}{\boldsymbol{\alpha}}_{kj}=\left[1,1,{\boldsymbol{\alpha}}_{k-1,j-1},{\boldsymbol{\alpha}}_{k-1,j},{\boldsymbol{\alpha}}_{k-1,j-1}+1,{\boldsymbol{\alpha}}_{k-1,j}+1\right]\\ {}{\boldsymbol{\beta}}_{kj}=\left[0,1,{\boldsymbol{\beta}}_{k-1,j-1},{\boldsymbol{\beta}}_{k-1,j},{\boldsymbol{\beta}}_{k-1,j-1},{\boldsymbol{\beta}}_{k-1,j}\right]\\ {}{\boldsymbol{\gamma}}_{kj}=\left[{\delta}_{k-1,j-1},-{\delta}_{k-1,j},\varDelta {\boldsymbol{\gamma}}_{k-1,j-1},{\scriptscriptstyle \frac{1-\varDelta }{n}}{\boldsymbol{\gamma}}_{k-1,j},{\boldsymbol{\phi}}_{k-1,j-1},-{\boldsymbol{\phi}}_{k-1,j}\right]\\ {}{\delta}_{kj}=\varDelta {\delta}_{k-1,j-1}+{\scriptscriptstyle \frac{1-\varDelta }{n}}{\delta}_{k-1,j}+{\scriptscriptstyle \frac{1}{n^{k-j}}}\left({\varepsilon}_{k-1,j-1}-{\varepsilon}_{k-1,j}\right)\\ {}\kern1.69em -{\boldsymbol{\phi}}_{k-1,j-1}^T{\left(-{\boldsymbol{\beta}}_{k-1,j-1} \log (n)\right)}^{\circ {\boldsymbol{\alpha}}_{k-1,j-1}+1}+{\boldsymbol{\phi}}_{k-1,j}^T{\left(\left(1-{\boldsymbol{\beta}}_{k-1,j}\right) \log (n)\right)}^{\circ {\boldsymbol{\alpha}}_{k-1,j}+1}\\ {}{\varepsilon}_{k\ j}=\left(1-\varDelta \right)\left({\varepsilon}_{k-1,j}-{\varepsilon}_{k-1,j-1}\right)+n{\varepsilon}_{k-1,j},\;\end{array} $$

*with shorthand*

$$ {\boldsymbol{\phi}}_{k\hbox{'},j\hbox{'}}\equiv {\boldsymbol{\gamma}}_{k\hbox{'},j\hbox{'}}\circ /\left({\boldsymbol{\alpha}}_{k\hbox{'},j\hbox{'}}+1\right), $$

*and, for j* = *k*,

10 $$ \begin{array}{l}{\boldsymbol{\alpha}}_{kk}=\left[1,{\boldsymbol{\alpha}}_{k-1,k-1},{\boldsymbol{\alpha}}_{k-1,k-1}+1\right]\\ {}{\boldsymbol{\beta}}_{kk}=\left[0,{\boldsymbol{\beta}}_{k-1,k-1},{\boldsymbol{\beta}}_{k-1,k-1}\right]\\ {}{\boldsymbol{\gamma}}_{kk} = \left[{\delta}_{k-1,k-1},\varDelta {\boldsymbol{\gamma}}_{k-1,k-1},{\boldsymbol{\phi}}_{k-1,k-1}\right]\\ {}{\delta}_{kk}=\varDelta {\delta}_{k-1,k-1}+{\varepsilon}_{k-1,k-1}\\ {}{\varepsilon}_{kk}=\left(1-\varDelta \right)\left(1-{\varepsilon}_{k-1,k-1}\right).\;\end{array} $$

In the above expressions, division (*γ* divided by ***α***+1) and exponentiation (***β*** to the power ***α***+1) are to be interpreted element-wise (hence the “∘”) and [1, 1, ***α***_*k*−1,*j*−1_, …] stands for the concatenation of elements and vectors into a new (longer) vector. The update equations can be initialized by setting

11 $$ {\varepsilon}_{k\hbox{'}0}={n}^{k\hbox{'}},\kern0.37em {\delta}_{k\hbox{'}0}=0,\kern0.24em \mathrm{and}\kern0.5em {\boldsymbol{\alpha}}_{k\hbox{'},0}={\boldsymbol{\beta}}_{k\hbox{'},0}={\boldsymbol{\gamma}}_{k\hbox{'},0}=\mathbf{0}, $$

for all 0 ≤ *k*' ≤ *k*.

From the updates it can be seen that each *α*_*k*,*j*,*m*_ ∈ {1, …, *k*} and each *β*_*k*,*j*,*m*_ ∈ {0, 1}. So, at most there will be 2 *k* unique combinations of *α* and *β* values. In an actual implementation, with every update we first compute and concatenate all *α’s* and *β’s* and then confine them to unique combinations by adding the *γ* coefficients that correspond to the same combination.

To compute ${\tilde{G}}_k\left(\boldsymbol{\rho} \right)$ for the whole range of rank products ***ρ*** at once, we first compute the set of corresponding intervals labelled by **j**. For all *j* ∈ **j** we then need to calculate the corresponding *θ*_*kj*_. We can do this recursively or using for-loops. When doing this recursively, it is wise to keep track of the parameters that already have been computed to prevent repetitive calculations. See Algorithm 1 in the Additional file 5. When using for-loops, following the same line of reasoning as suggested by Figure 1, we have an outer loop with *j*' running from 0 to max(**j**) (from left to right) and an inner loop with *k*' running from *j*' to max(*k* − min(**j**) + *j*', *k*) (from top to bottom). Alternatively, we can have an outer loop with *k*' running from 0 to *k* (from top to bottom) and *j*' from max(*k*' − *k* + min(**j**), 0) to min(*k*', max(**j**)) (from left to right). This latter ordering is taken in Algorithm 2 in Additional file 5. The solution for each *ρ* then follows by computing ${\tilde{G}}_{kj}\left(\rho \right)$ from (8), with *j* labelling the interval containing *ρ*. Algorithm 1 is implemented in R (R Core Team [14]) and the R code is published in Additional file 6 and is available at http://www.ru.nl/publish/pages/726696/rankprodbounds.zip.

### Exact calculation and gamma approximation

The exact *p*-values may be obtained by a brute force search using the discrete recursion (1). An alternative method, proposed by Eisinga *et al.* [11], is to use number theory to obtain a combinatorial exact expression for calculating the discrete probability distribution of the rank product statistic. The distribution is asymmetric (i.e., positively skewed) and in determining the *p*-value, all probabilities need to be calculated, from the smallest rank product possible, with *ρ* = 1, to the rank product value of interest. This implies that the exact statistical significance of large rank products may take unacceptably long amounts of time to compute [11,15,16].

In [13], Koziol argues that under the null hypothesis for experiment *i* the *p*-values *r*_*i*_/(*n* + 1) are approximately uniformly distributed on the interval [0,1]. As the probability distribution of the negative log-transformed *p*-values is given by the exponential distribution with scale parameter 1, the negative sum of the log-transformed *p*-values over *k* independent experiments has a Gamma(*k*,1) distribution (see also Pounds and Cheng [17]). This approach is equivalent to Fisher’s [18] method of combining *p*-values over independent tests. As illustrated below, the assumption that the distribution of the *p*-values is uniform on the continuous interval [0,1], when in fact it is uniform on the discrete set {1/(*n* + 1), 2/(*n* + 1), …, *n*/(*n* + 1)}, leads to substantial deviations from the right tail of the true distribution.

## Results and discussion

### Time performance and accuracy

The R program computes the bounds and the geometric mean *p*-value approximation at a very fast speed. For example, it takes approximately 2 milliseconds to calculate the upper bound *p*-value of any rank product *ρ* in the range 1 to *n*^*k*^, for *n* = 10000 and *k* = 4, on a HP desktop computer using the interpreted R language running under Windows 7 with an Intel Core i7 CPU at 2.9 GHz. It takes twice as much time to calculate the geometric mean *p*-value approximation. Unlike exact calculation, the algorithm’s computational time is almost unrelated to the value of rank product *ρ.*

**Figure 2:**
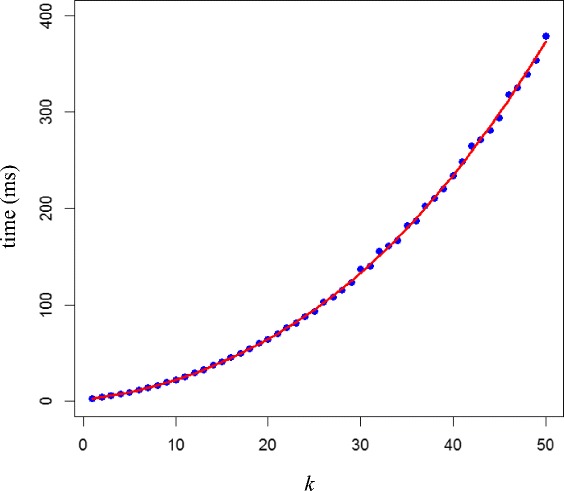
**Computation time (in milliseconds) for calculating 10000 upper bound** ***p*** **-values for** ***n*** **= 10000 and** ***k*** **= 2, …, 50.**

To examine the effect of the number of experiments *k* on the algorithm’s running time, we generated 10000 random draws from the discrete uniform distribution on [1, *n*^*k*^] and calculated the upper bounded *p*-value of the simulated rank products, for *n* = 10000 and *k* = 2, …, 50. Figure 2 plots the computation time (in milliseconds) for the calculation of 10000 *p*-values and a third-order polynomial fitted line.

The figure indicates that computation time is no limiting factor when it comes to approximate *p*-value calculation of rank products, even for very demanding problems. Running time increases polynomially (of maximum order 3) with increasing *k.* Also, the time needed to do the same calculation for much larger *n* is similar to the time figures shown in the plot, as the algorithm’s computational time is not only virtually unrelated to rank product *ρ*, but also unaffected by *n*. This implies that the proposed calculation method should work well with all sample and replicate sizes typically encountered in postgenomic molecular profiling experiments.

**Figure 3:**
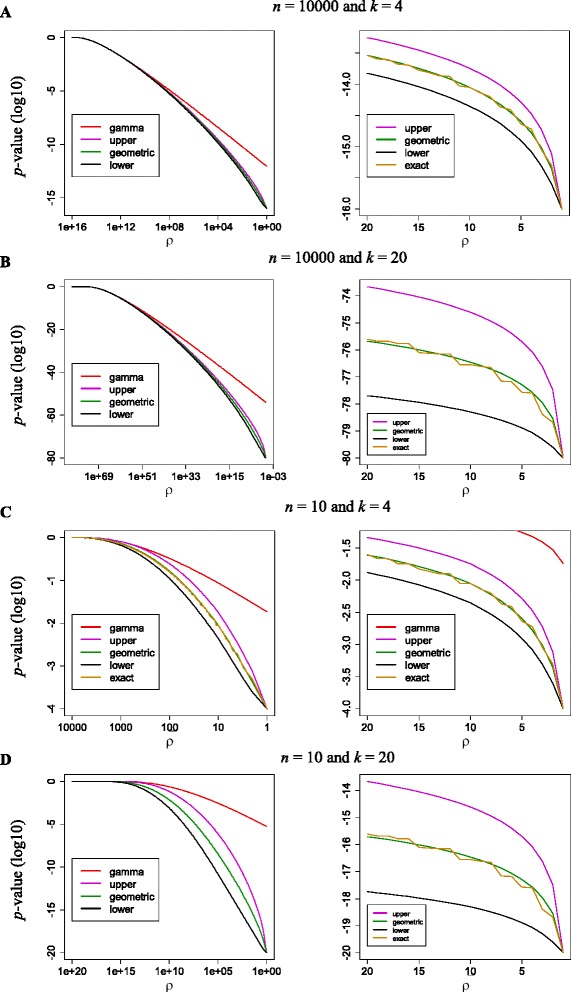
**Bounds and approximations of** ***p*** **-value distribution. (A)** Strict bounds and approximations (geometric mean of upper and lower bound, and gamma) for *n* = 10000 molecules and *k* = 4 experiments, on the left-hand side over the whole range of rank products, on the right-hand side for small rank products only (gamma approximation is outside the figure). It can be seen that, on the log scale, the bounds are very tight. Zooming in on small rank products, the bounds are on average about a factor 2.5 off (i.e., higher/lower than the exact *p*-value), yet the geometric mean approximation is still very close to the exact *p*-value. **(B)** Same as **(A)**, but for *n* = 10000 and *k* = 20. The curve on the left looks more or less the same but, as is best seen on the right, the bounds are much further off (almost a factor 1000). **(C)** Same as **(A)**, but for *n* = 10 and *k* = 4. The curve on the left may look worse, but that is mainly because of the scaling of the *y*-axis. Relatively speaking, the bounds are still on average about a factor 2.5 off. **(D)** Same as **(A)**, but for *n* = 10 and *k* = 20. With very small *n* and relatively large *k*, we get the worst of both worlds.

To assess numerical accuracy, the entire *p*-value distribution was obtained for both large and small values of *n* and *k* (i.e., *n* = 10,10000 and *k* = 4,20)*.* Figure 3 displays the gamma approximation, the upper and lower bounds, and the geometric mean *p*-value approximation. The exact *p*-values are reported only for small values of *ρ* (right-hand panels of Figure 3) and for the entire range of rank products of the smallest *n* and *k* (left-hand panel of Figure 3C). Exact *p*-value calculation of the entire distribution is computationally unmanageable (or at least extremely time consuming) for the other values of *n* and *k*. As can be seen in Figure 3A, the upper and lower bounds are rather tight. Relatively speaking, i.e., on a logarithmic scale, they are most tight for large rank products. For small rank products they are in this case (*n* = 10000 and *k* = 4) at most a factor 3 off, that is higher/lower than the exact *p*-value. The approximation obtained by taking the geometric mean of the upper and lower bound is seen to be very accurate.

Trying different values of *n* and *k*, the curves look extremely similar when we plot them over the entire range of rank products, that is, for log-transformed *p*-values, between − *k*log*n* and 0. The range between the log upper bound and the log lower bound is more or less independent of *n* and increases roughly linear with *k,* but then so does the range of log *p*-values. With increasing *n*, the range of log *p*-values does increase logarithmically with *n*, where the range between upper and lower bound remains about constant (see Figure 3C for *n* = 10 and *k* = 4). This makes that curves for large *n* look most impressive in the sense of displaying tight bounds. Results for small *n* and large *k* are least impressive (see Figure 3D for *n* = 10 and *k* = 20). In any case, excluding extremely large rank products, the upper bounds are always orders of magnitude better than the gamma approximation. The latter assumes a continuous distribution and this assumption is too strong for the analysis of discrete rank products.

When trying to find an even better approximation or bound for *G*_*k*_(*ρ*), one option is to use the continuous approximation scheme to compute ${\tilde{G}}_{k-1}\left(\rho \hbox{'}\right)$ for all *ρ*' ≤ *ρ* and then apply the discrete recursion (1) to arrive at better ${\tilde{G}}_k\left(\rho \right).$ Initial attempts revealed that this indeed yields somewhat tighter bounds (e.g., a factor 1.5 off instead of 2.5) and a more accurate approximation, but not to the extent that it seems worth the computational effort.

### Application

To illustrate our method in a real-world application, gene expression data on human aging were obtained from Van den Akker *et al*. [19], available at http://onlinelibrary.wiley.com/doi/10.1111/acel.12160/suppinfo [Supplementary Table S2]. The data set contains the statistical results for 9047 unique genes (expressed in 2539 individuals) from four different studies. The authors employed rank product analysis to identify genes consistently up- or down-regulated with age across the four data sets. Table 1 displays the top 25 genes having increased expression with age.

**Table 1:** **Top-25 age-associated genes with increased expression level (Van den Akker** ***et al.*** [19]**)**

**Symbol**	**GeneID**	***ρ***			***p*** **-value**		
			**Exact**	**Gamma**	**Upper bound**	**Geometric mean**	**Lower bound**
GPR56	9289	9282	2.645 × 10^−10^	5.255 × 10^−09^	3.888 × 10^−10^	2.709 × 10^−10^	1.887 × 10^−10^
HF1	3075	48576	2.074 × 10^−09^	2.296 × 10^−08^	2.873 × 10^−09^	2.117 × 10^−09^	1.559 × 10^−09^
SYT11	23208	57600	2.550 × 10^−09^	2.671 × 10^−08^	3.510 × 10^−09^	2.601 × 10^−09^	1.927 × 10^−09^
ARP10	164668	179400	9.817 × 10^−09^	7.297 × 10^−08^	1.303 × 10^−08^	1.000 × 10^−08^	7.680 × 10^−09^
B3GAT1(CD57)	27087	278460	1.635 × 10^−08^	1.075 × 10^−07^	2.142 × 10^−08^	1.666 × 10^−08^	1.295 × 10^−08^
SLC1A7	6512	483780	3.078 × 10^−08^	1.746 × 10^−07^	3.970 × 10^−08^	3.135 × 10^−08^	2.476 × 10^−08^
IFNG	3458	1594440	1.171 × 10^−07^	4.953 × 10^−07^	1.465 × 10^−07^	1.192 × 10^−07^	9.697 × 10^−08^
DSCR1L1	10231	2004864	1.507 × 10^−07^	6.046 × 10^−07^	1.874 × 10^−07^	1.533 × 10^−07^	1.254 × 10^−07^
ARK5	9891	2726880	2.110 × 10^−07^	7.898 × 10^−07^	2.605 × 10^−07^	2.146 × 10^−07^	1.768 × 10^−07^
PIG13	81563	3549314	2.809 × 10^−07^	9.927 × 10^−07^	3.448 × 10^−07^	2.857 × 10^−07^	2.367 × 10^−07^
SPUVE	11098	3880576	3.093 × 10^−07^	1.072 × 10^−06^	3.789 × 10^−07^	3.146 × 10^−07^	2.612 × 10^−07^
PDGFRB	5159	4294368	3.451 × 10^−07^	1.171 × 10^−06^	4.217 × 10^−07^	3.509 × 10^−07^	2.920 × 10^−07^
EDG8	53637	5083584	4.137 × 10^−07^	1.355 × 10^−06^	5.037 × 10^−07^	4.207 × 10^−07^	3.513 × 10^−07^
MARLIN1	152789	5505984	4.507 × 10^−07^	1.451 × 10^−06^	5.477 × 10^−07^	4.582 × 10^−07^	3.833 × 10^−07^
TGFBR3	7049	8081700	6.784 × 10^−07^	2.021 × 10^−06^	8.176 × 10^−07^	6.896 × 10^−07^	5.815 × 10^−07^
GZMB	3002	9886240	8.396 × 10^−07^	2.404 × 10^−06^	1.008 × 10^−06^	8.533 × 10^−07^	7.227 × 10^−07^
DEFA3	1168	9980528	8.481 × 10^−07^	2.423 × 10^−06^	1.018 × 10^−06^	8.619 × 10^−07^	7.301 × 10^−07^
KRT1	3848	11787930	1.010 × 10^−06^	2.796 × 10^−06^	1.208 × 10^−06^	1.027 × 10^−06^	8.728 × 10^−07^
CX3CR1	1524	12060288	1.035 × 10^−06^	2.851 × 10^−06^	1.237 × 10^−06^	1.052 × 10^−06^	8.944 × 10^−07^
STYK1	55359	14337372	1.241 × 10^−06^	3.308 × 10^−06^	1.477 × 10^−06^	1.260 × 10^−06^	1.076 × 10^−06^
ADRB2	154	16272900	1.416 × 10^−06^	3.687 × 10^−06^	1.681 × 10^−06^	1.438 × 10^−06^	1.231 × 10^−06^
GAF1	26056	35217600	3.138 × 10^−06^	7.128 × 10^−06^	3.667 × 10^−06^	3.186 × 10^−06^	2.769 × 10^−06^
CTSL	1514	38246400	3.414 × 10^−06^	7.647 × 10^−06^	3.982 × 10^−06^	3.465 × 10^−06^	3.016 × 10^−06^
GFI1	2672	56960480	5.107 × 10^−06^	1.072 × 10^−05^	5.907 × 10^−06^	5.183 × 10^−06^	4.547 × 10^−06^
TTC38	55020	59340600	5.322 × 10^−06^	1.110 × 10^−05^	6.150 × 10^−06^	5.400 × 10^−06^	4.742 × 10^−06^

We obtained the exact *p*-values and, ideally, one should use these values in correcting for multiple testing as they are the gold standard in the sense that the sampling distribution is known exactly. Only by deciding to accept or reject the null on the basis of exact *p*-values are we guaranteed to be protected from Type-1 errors at the desired significance level. However, it takes considerable amounts of time to calculate the *p*-value for the gene listed in the bottom of Table 1 (approximately 120 minutes) and it is (by far) not feasible to obtain the exact *p*-values of the largest rank products on a timely enough basis. The strict upper and lower bounds, however, perform well in the sense that the limits are narrow and the bias is tiny. Although the geometric mean *p*-value approximation provides no absolute guarantee to protection from Type-1 errors, the estimates and the exact probabilities are seen to be very close. The gamma distribution is seen to produce rather inaccurate approximate results.

Bonferroni corrections are one approach for controlling the experiment-wide false positive rate (*π*) by specifying what *α* value should be used for each individual test, taking *α* = *π*/*n*. For the current study, *π* = 0.05 gives *α* = 0.05/9047 ≈ 5.526 × 10^−6^. We declare a test (i.e., gene) to be significant if *p* ≤ *α*.

The results for both up- and down-regulated genes are shown in the left panel of Table 2. Under a strict Bonferroni correction, we reject the null hypothesis of no differential expression with associated exact *p*-value for 25 up- and 42 down-regulated genes. The geometric mean *p*-value approximation produces results identical to the exact method. The asymptotic gamma approximation is too conservative in that it tends to understate the evidence against the null hypothesis. While reducing the number of false positives, it also reduces the number of true discoveries, especially for down-regulated genes. The Bonferroni method applied to the gamma *p*-values declared 30 genes to be significant, instead of 42.

**Table 2:** **Number of genes called significant according to Bonferroni correction and FDR** ***q*** **-values**

	**Bonferroni correction**		***q*** **-value**	
		**< 0.001**	**< 0.01**	**< 0.05**
**Up-regulated genes**				
Exact	25			
Gamma	21	14	40	112
Upper bound	23	21	57	122
Geometric mean	25	21	58	129
Lower bound	26	21	59	131
**Down-regulated genes**				
Exact	42			
Gamma	30	23	69	140
Upper bound	42	39	74	143
Geometric mean	42	42	74	154
Lower bound	43	43	75	157

The traditional Bonferroni correction may be too stringent in postgenomic multiple testing, where the number of molecules profiled in parallel is very large, and falsely detecting a small number of molecules as differently expressed will usually not be a serious problem if the majority of significant molecules are properly selected. A less stringent method is to estimate the FDR for the entire collection of *p*-values, defined as the expected number of false positives amongst the molecules selected as significantly differentially expressed, described in detail in Storey [20] and Storey and Tibshirani [9]. We obtained the FDR adjusted *p*-values, i.e., *q*-values, for all approximate *p*-value estimates, using Storey’s R program Q-value (with the bootstrap estimator). The estimated *q*-value for any particular test is a function of the *p*-value for that test and the distribution of the entire set of *p*-values. As it utilizes information from all the *p*-values at once, it is impossible to obtain *q*-values based on the exact probabilities. The right panel of Table 2 presents the number of significant calls for various thresholds by *p*-value approximation method. As can be seen, about [(57–40)/57 × 100=] 30% of the differentially expressed up-regulated genes selected using the upper bounded *p*-values at a *q*-value of 0.01, were not detected by the overly conservative gamma approach.

## Conclusions

In replicated molecular profiling experiments, where large numbers of molecules are simultaneously tested, accurately estimated *p*-values are essential for making justified, reproducible decisions about which molecules to consider as significantly differentially expressed in the downstream analysis. We provide a tailor-made solution to calculate strict bounds and accurate approximate *p*-values for rank product analysis of postgenomic molecular profiling data. The proposed algorithm runs very fast and gives a slightly conservative upper bound protecting against false positives and a close approximate estimate of the true *p*-values.

Over the past decade, the rank product method, developed originally for the analysis of microarray datasets, has found widespread use in various settings such as proteomics [6,7], metabolomics [8], RNAi screening [21], meta-analysis [4,15,22], and classification [23]. However, its application has been restricted to medium sample and replicate sizes due to an intensive permutation test used to calculate significance. The algorithm presented here can provide an order of magnitude increase in throughput as compared with permutation testing. It also allows researchers to explore new application domains with even larger multiple testing issue, e.g., in large genetics studies with millions of markers or RNAseq analyses where the number of studies transcripts is larger than the number of genes or in applications to image analysis.

### Software availability

The R code is also freely available at http://www.ru.nl/publish/pages/726696/rankprodbounds.zip.

## Additional files

Additional file 1:
**Proof of Theorem 1.**
Additional file 2:
**Proof of Theorem 2.**
Additional file 3:
**Proof of Theorem 3.**
Additional file 4:
**Proof of Theorem 4.**
Additional file 5:
**Pseudo code.** A pdf file providing the pseudo code of two algorithms. Additional file 6:
**Rankprodbounds.** A .zip file providing the code of the algorithm implemented in R.
