# Supplementary material for: A fast algorithm for determining bounds and accurate approximate p-values of the rank product statistic for replicate experiments
Source: BMC Bioinformatics. 2014 Nov 21;15(1):367. doi: 10.1186/s12859-014-0367-1 (PMC4245829; doi:10.1186/s12859-014-0367-1)
Supplement: Additional file 1: — Proof of Theorem 1. [file 12859_2014_367_MOESM1_ESM.doc]

Additional file 1

*Proof of Theorem 1.* Since

$$G_{k-1}(\rho/r_{1})\geq\int_{r_{ 1}}^{r_{ 1}+1} G_{k-1}(\rho/r) dr\geq G_{k-1}(\rho/(r_{1}+1)),$$

we have

$$\sum_{r_{1}=1}^{\min(\rho,n)-1} G_{k-1}(\rho/r_{1})\geq\int_{1}^{\min(\rho,n)} G_{k-1}(\rho/r) dr\geq\sum_{r_{ 1}=2}^{\min(\rho,n)} G_{k-1}(\rho/r_{1}),$$

and hence

$$\begin{matrix} \int_{1}^{\min(\rho,n)} G_{k-1}(\rho/r) dr+G_{k-1}(\max(1,\rho/n))\leq\\ \sum_{r_{ 1}=1}^{\min(\rho,n)-1} G_{k-1}(\rho/r_{1})+G_{k-1}(\rho/\min(\rho,n))=G_{k}(\rho)= \\ =G_{k-1}(\rho)+\sum_{r_{ 1}=2}^{\min(\rho,n)} G_{k-1}(\rho/r_{1})\leq G_{k-1}(\rho)+\int_{1}^{\min(\rho,n)} G_{k-1}(\rho/r) dr. \end{matrix}$$

It now follows by induction that the solutions of the recursions (2) and (3) indeed give an upper and lower bound, respectively. For example, for the upper bound, suppose that indeed $\bar{G}_{k-1}(\rho)\geq G_{k-1}(\rho)\text{,}$ which is by definition the case for $k=0:\bar{G}_{0}(\rho)=G_{0}(\rho).$ Then

$$\begin{matrix} \bar{G}_{k}(\rho)=\bar{G}_{k-1}(\rho)+\int_{1}^{\min(\rho,n)} \bar{G}_{k-1}(\rho/r) dr\geq\\ G_{k-1}(\rho)+\int_{1}^{\min(\rho,n)} G_{k-1}(\rho/r) dr \\ \geq G_{k-1}(\rho)+\sum_{r_{ 1}=2}^{\min(\rho,n)} G_{k-1}(\rho/r)=G_{k}(\rho). \end{matrix}$$

Similarly, for the lower bound: if $\underline{G}_{k-1}(\rho)\geq G_{k-1}(\rho)$, with $\underline{G}_{0}(\rho)=G_{0}(\rho)\text{,}$ then

## 
