# Supplementary material for: A fast algorithm for determining bounds and accurate approximate p-values of the rank product statistic for replicate experiments
Source: BMC Bioinformatics. 2014 Nov 21;15(1):367. doi: 10.1186/s12859-014-0367-1 (PMC4245829; doi:10.1186/s12859-014-0367-1)
Supplement: Additional file 2: — Proof of Theorem 2. [file 12859_2014_367_MOESM2_ESM.doc]

Additional file 2

*Proof of Theorem 2*. We start from the recursion (4) and consider a value of $\rho$ within the *j*th interval, i.e., such that $n^{k-j}\leq\rho<n^{k-j+1}.$ By definition we then have $\tilde{G}_{k}(\rho)=\tilde{G}_{kj}(\rho).$

We first consider the case $1\leq j\leq k-1\text{,}$ for which we have $\min(\rho,n)=n$ and $\text{max}(1,\rho/n)=\rho/n.$ We split up the integral in (4) into two terms: for small *r*, the argument $\rho/r$ will still be between $n^{k-j}\leq\rho/r<n^{k-j+1}$ and we need to substitute $G_{k-1,j-1}(\rho/r).$ For larger *r*, the argument $\rho/r$ falls in the interval $n^{k-1-j}\leq\rho/r<n^{k-j}$ and we require $G_{k-1,j}(\rho/r).$ Similarly, we have $G_{k-1}(\rho)=G_{k-1,j-1}(\rho)$ and $G_{k-1}(\rho/n)=G_{k-1,j}(\rho/n).$ Substituting the above into (4), we obtain (6).

For *j=k*, we have $\min(\rho,n)=\rho$ and $\max(1,\rho/n)=1.$ We still have $G_{k-1}(\rho)=G_{k-1,k-1}(\rho)\text{,}$ but now we need $\tilde{G}_{k-1}(1).$ Considering the recursion (4) for $\rho=1\text{,}$ we obtain $\tilde{G}_{k}(1)=\tilde{G}_{k-1}(1)=\tilde{G}_{0}(1)=1\text{,}$ as one would expect. Substituting the above into (4), we obtain (7).
