# Supplementary material for: A fast algorithm for determining bounds and accurate approximate p-values of the rank product statistic for replicate experiments
Source: BMC Bioinformatics. 2014 Nov 21;15(1):367. doi: 10.1186/s12859-014-0367-1 (PMC4245829; doi:10.1186/s12859-014-0367-1)
Supplement: Additional file 3: — Proof of Theorem 3. [file 12859_2014_367_MOESM3_ESM.doc]

Additional file 3

*Proof of Theorem 3*. The proof follows by induction. It is clear that $\tilde{G}_{k0}$ itself can be written in the form (8). Plugging the conjectured form (8) into the right-hand side of the recursions (6) and (7) we obtain three types of integrals: for the constant $\varepsilon\text{-}$term, for the $\delta\text{-}$term proportional to $\rho\text{,}$ and for the $\gamma\text{-}$terms proportional to $\Psi(\rho;\alpha,\lambda).$ The $\gamma\text{-}$terms give integrals of the form

(12)

and

(13)

Both results are of the form (8). The integration that results from the $\delta\text{-}$term proportional to $\rho$ boils down to a special case of the above integrals, since $\rho=\Psi(\rho;0,1)\text{:}$

(14)

and

(15)

Since the integrals limits are all either constant or proportional to $\rho\text{,}$ integration over the constant $\varepsilon\text{-}$term yields terms that are themselves constant or proportional to $\rho.$ Finally, it is easy to check that the terms that are not integrated over, in particular $\tilde{G}_{k-1,j}(\rho/n)$ in (6), also obey the form (8). Summarizing, all terms on the right-hand side of (6) and (7) can be written in the form (8). Finally, we note that the function space defined by (8) is closed under addition and thus the sum of such terms can again be written in the same form.
