# Supplementary material for: A fast algorithm for determining bounds and accurate approximate p-values of the rank product statistic for replicate experiments
Source: BMC Bioinformatics. 2014 Nov 21;15(1):367. doi: 10.1186/s12859-014-0367-1 (PMC4245829; doi:10.1186/s12859-014-0367-1)
Supplement: Additional file 4: — Proof of Theorem 4. [file 12859_2014_367_MOESM4_ESM.doc]

Additional file 4

*Proof of Theorem 4*. Given the integrals in the previous proof, deriving the update equations is just a matter of proper bookkeeping. Here we indicate where the various terms come from without writing out the equations.

The first two terms in the update equations (9) of $\alpha\text{,}$ $\beta\text{,}$ and $\gamma$ for $1\leq j\leq k-1$ stem from the integrals (14) and (15) over terms in $\tilde{G}_{k-1,j-1}$ and $\tilde{G}_{k-1,j}\text{,}$ respectively, proportional to $\rho.$ The third and fourth term are essentially copied from the terms $\Delta\tilde{G}_{k-1,j-1}$ and $(1-\Delta)\tilde{G}_{k-1,j}$ in (6) that are not integrated over. The fifth and sixth terms go back to the integrals (12) and (13) over the $\Psi\text{-}$terms in $\tilde{G}_{k-1,j-1}$ and $\tilde{G}_{k-1,j}\text{,}$ respectively. The first two terms in the update equation of $\delta$ are again copies of the terms that are not integrated over, the third and fourth term follow by integrating the constant term, and the fifth and sixth term stem from the integrals (12) and (13). The update equation for $\varepsilon$ combines the constant terms that are and those that are not integrated over.

The update equations (10) for $j=k$ follow the same line of reasoning, except that now there is only a single integral. In essence the updates of $\alpha\text{,}$ $\beta\text{,}$ $\gamma\text{,}$ and $\delta$ for $k=j$ are the same as those for $k<j\text{,}$ except that we can ignore all terms involving indices $(k-1,k).$ From the update equation for $\beta_{kk}$ we realize that all elements from $\beta_{k'k'}$ remain zero, so that also the term proportional to $\gamma_{k-1,k-1}$ in the update for $\delta_{kk}$ drops out. The update equation for $\varepsilon$ again combines the constant terms that are and those that are not integrated over.
