# Supplementary material for: A fast algorithm for determining bounds and accurate approximate p-values of the rank product statistic for replicate experiments
Source: BMC Bioinformatics. 2014 Nov 21;15(1):367. doi: 10.1186/s12859-014-0367-1 (PMC4245829; doi:10.1186/s12859-014-0367-1)
Supplement: Additional file 5: — Pseudo code. A pdf file providing the pseudo code of two algorithms. [file 12859_2014_367_MOESM5_ESM.pdf]

A fast algorithm for determining bounds and accurate approximate *p*-values of the rank product statistic for replicate experiments

by Heskes, Eisinga & Breitling, *BMC Bioinformatics*, 2014

Algorithm 1. Recursive algorithm for approximating *p*-values over a range of rank products $\boldsymbol{\rho}$

function Rankproductbounds $(\boldsymbol{\rho}, n, k, \Delta)$
  $\mathbf{j} = \left\lceil -\frac{\log(\rho)}{\log(n)} + k \right\rceil$
  for $j = \min(\mathbf{j}) \rightarrow \max(\mathbf{j})$ **do**
    $\boldsymbol{\theta} = \mathbf{UpdateParam}(\boldsymbol{\theta}, n, k, j, \Delta)$
  Compute $\tilde{G}_{k,\mathbf{j}}(\boldsymbol{\rho})$ from (8)
  return $\frac{\tilde{G}_{k,\mathbf{j}}(\rho)}{n^k}$
function UpdateParam $(\boldsymbol{\theta}, n, k, j, \Delta)$
  if not yet computed $\theta_{k,j}$ **then**
    if $j = 0$ **then**
      Initialize $\theta_{k,0}$ as in (11)
    else
      $\boldsymbol{\theta} = \mathbf{UpdateParam}(\boldsymbol{\theta}, n, k-1, j-1, \Delta)$
      if $j < k$ **then**
        $\boldsymbol{\theta} = \mathbf{UpdateParam}(\boldsymbol{\theta}, n, k-1, j, \Delta)$
        Compute $\theta_{k,j}$ from $\theta_{k-1,j-1}$ and $\theta_{k-1,j}$ as in (9)
      else
        Compute $\theta_{k,k}$ from $\theta_{k-1,k-1}$ as in (10)
    return $\boldsymbol{\theta}$

Algorithm 2. Algorithm for approximating *p*-values over a range of rank products $\boldsymbol{\rho}$ using for-loops

function Rankproductbounds $(\boldsymbol{\rho}, n, k, \Delta)$
  $\mathbf{j} = \left\lceil -\frac{\log(\rho)}{\log(n)} + k \right\rceil$
  for $k' = 0 \rightarrow k$ **do**
    for $j' = \max(k' - k + \min(\mathbf{j}), 0) \rightarrow \min(k', \max(\mathbf{j}))$ **do**
      if $j' = 0$ **then**
        Initialize $\theta_{k',0}$ as in (11)
      else
        if $j' < k'$ **then**
          Update $\theta_{k',j'}$ as in (9)
        else
          Update $\theta_{k',k'}$ as in (10)
        Combine all unique combinations of $\boldsymbol{\alpha}_{k',j'}$ and $\boldsymbol{\beta}_{k',j'}$ by adding their $\boldsymbol{\gamma}_{k',j'}$
  Compute $\tilde{G}_{k,\mathbf{j}}(\boldsymbol{\rho})$ from (8)
  return $\frac{\tilde{G}_{k,\mathbf{j}}(\rho)}{n^k}$
